# Assessing the impact of urban greenspace on physical health: An empirical study from Southwest China

**DOI:** 10.3389/fpubh.2023.1148582

**Published:** 2023-03-21

**Authors:** Barnabas C. Seyler, Han Luo, Xiuli Wang, Sophia Zuoqiu, Yao Xie, Yuan Wang

**Affiliations:** ^1^Department of Environment, Sichuan University, Chengdu, China; ^2^Shude International, Chengdu Shude High School, Chengdu, China; ^3^School of Software Engineering, Chengdu University of Information Technology, Chengdu, China; ^4^Sichuan Province Informationization Application Support Software Engineering Technology Research Center, Chengdu, China; ^5^West China School of Public Health, Sichuan University, Chengdu, China; ^6^State Key Laboratory of Marine Pollution, Department of Chemistry, City University of Hong Kong, Kowloon, Hong Kong SAR, China

**Keywords:** greenspace indicator, hospitalization rate, landscape heterogeneity, respiratory disease, urban ratio

## Abstract

**Introduction:**

As the world becomes increasingly urbanized and human-nature contact declines, urban greenspace's impact on human health has garnered growing interest across academic disciplines. Various definitions and multiple indicators of greenspace have been utilized, with most studies finding an overall positive association between greenspace and health. Nevertheless, studies directly comparing how different greenspace indicators impact different disease types have been limited. Moreover, to verify the robustness of conclusions drawn, studies should compare multiple measures of greenspace across various spatial scales. Thus, a more comprehensive analysis is necessary to help inform future study design, especially in determining which greenspace indicators would be most useful in data-limited areas.

**Methods:**

Chengdu, the capital city of Sichuan Province, is West China's largest and most urban city, being typical of other large cities in lower to middle-income countries (LMICs). With twenty county-level jurisdictions spanning various degrees of urbanization, Chengdu's landscape heterogeneity and large population make it ideal for studying greenspace's impact on public health. This study took Chengdu as a case study to assess the association and potential impact of three traditional measures of greenspace (Normalized Difference Vegetation Index, Enhanced Vegetation Index, and Fractional Vegetation Cover) and urban ratio (% of population being urban) on hospitalization rates and medical expenses paid for three major disease categories (circulatory system diseases, neoplasms, and respiratory system diseases).

**Results and discussion:**

We found greenspace did have a significant impact on public health, but this relationship differed by disease type. Greenspace exhibited significant positive association with respiratory diseases, but insignificant negative associations with the other disease categories. Urban ratio showed significant negative association with greenspace abundance. The higher the urban ratio (e.g., less greenspace), the more money was paid on medical expenses. This relationship was found not only in terms of urban ratio being positively correlated with medical expenses, but also in that all three greenspace indicators were negatively correlated with medical expenses. Consequently, in future health outcome studies, urban ratio could be an acceptable negative indicator of greenness in LMICs where urban ratio is likely to imply less greenness.

## 1. Introduction

As socio-demographic, economic, and modernization forces transform the Anthropocene landscape, human populations are increasingly concentrated in urban areas. According to the United Nations, more than 55% of the global population now lives in cities, with 68% projected to by 2050 (1). With higher population densities, the percent coverage of urban greenspace is much less than in rural areas. Consequently, the impact of greenspace on human wellbeing has attracted considerable attention across academic disciplines (2–8). Due to the interdisciplinary and multidisciplinary nature of this scholarship, various definitions and indicators of greenspace as well as human wellbeing have been utilized. Definitions of greenspace generally align with one of two broad characterizations: (1) an overarching concept of nature in which greenspace is essentially a “synonym of nature and antonym of urbanization,” or (2) urban vegetation itself, including various types of vegetated spaces found in urban environments (9).

Irrespective of how greenspace is characterized, in studies of human well-being the most frequently used indicator of greenspace abundance (i.e., local “greenness” or “exposure”) is Normalized Difference Vegetation Index (NDVI) (7, 10–13). NDVI assesses vegetation coverage by comparing near-infrared and red (visible) light reflected from the earth's surface during active photosynthesis (2, 7, 11). Because NDVI is calculated from satellite images, it allows assessment of greenspace exposure retrospectively (10). However, several weaknesses of NDVI have been identified, including its inability to differentiate degrees of vegetation coverage in high-biomass regions (e.g., subtropical and tropical rainforests), where NDVI signals become saturated, and its vulnerability to background noise (e.g., clouds and aerosols in the atmosphere, as well as reflective land surfaces) (14, 15). As a result, other indicators, such as Enhanced Vegetation Index (EVI) (13–15) and Fractional Vegetation Cover (FVC) have been utilized (16–18). As satellite imagery resolution increased, EVI was developed with several key improvements over NDVI. For example, EVI is more robust against background noise (e.g., atmospheric aerosols, surface reflectance, and soil color variations), but it is also more sensitive to high-density vegetation and canopy structural variety, making it more useful to facilitate comparisons across different spatial scales with non-uniform vegetation (14, 15). In contrast to NDVI and EVI, FVC is defined as the ratio between the above ground vegetation projected onto the surface to the total surface area (17, 19). Consequently, FVC (sometimes called “vegetation fraction”) is frequently used to monitor vegetation degradation, soil erosion, and desertification, as well as general trends in land surface processes, ecosystem function, and climate change (19).

Regardless of how greenspace is defined or measured, most studies have found a generally positive association with human wellbeing (3, 4, 7, 10, 20, 21). Various indicators for both physical and mental health have been used (10, 22, 23), and studies have documented both direct and indirect reasons for this positive association (5, 6, 12, 22). However, not all studies have found the positive association held true (24, 25), particularly when assessing the relationship on a city-wide scale (26). In addition, the standard deviation of greenness indicators such as NDVI can be understood to represent landscape heterogeneity, which can also have significant impacts on human health outcomes (27). Consequently, recent studies have emphasized the importance of simultaneously testing *multiple* indicators of greenspace abundance in studies on human health outcomes to verify the robustness of conclusions drawn across various spatial scales (2, 10, 23).

Moreover, ongoing urbanization and decreasing greenspace worldwide make these investigations increasingly important, especially outside of North America and Europe where studies predominate, but particularly in developing and lower to middle-income countries (LMICs) (4, 12, 28). As both the world's largest developing country and largest population (1), China is particularly well-suited to explore the impact of urban greenspace on human health. China has undergone rapid urbanization in recent decades, driven by world history's largest migration from rural-to-urban areas (29). As of 2018, 59% of China's 1.4 billion people (837 million) lived in urban areas, already representing the largest urban population (20% of global total), but 255 million additional urban dwellers are projected in China by 2050 (1). With acute land scarcity (30), urban greenspace scholarship in China has primarily focused on land use change and city planning policies (31–35). Studies have also sought to investigate how urban expansion affects greenspace exposure (36, 37), as well as quantify and evaluate the ecosystem functions and services provided by urban greenspaces (38–42). These studies have both direct and indirect implications for human health and wellbeing, but some significant studies specifically exploring greenspace's impact on population-level health outcomes have also been recently undertaken in China (43, 44).

**Figure 1:**
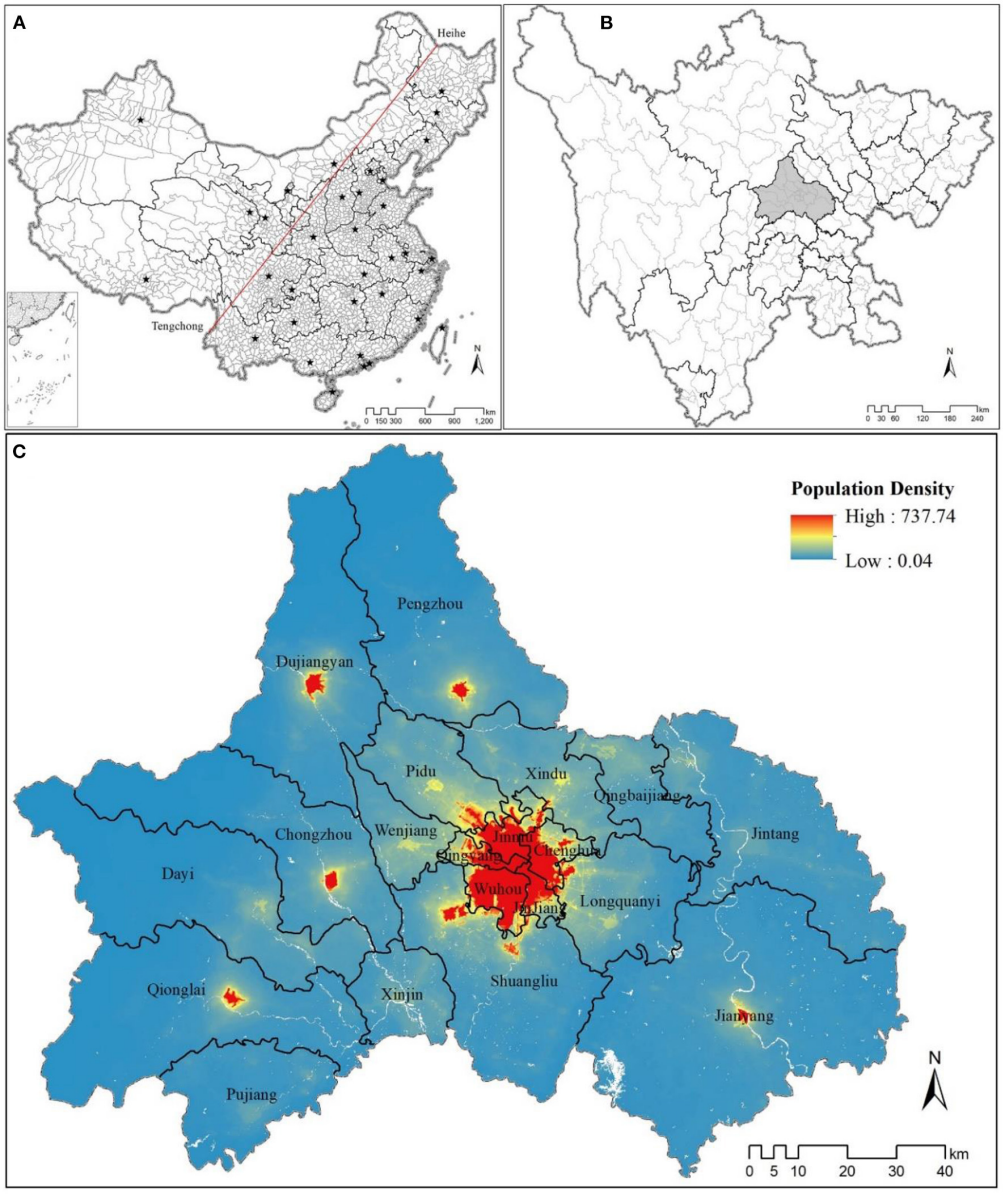
**(A)** Mainland China, with all provincial- and county-level jurisdictions, bisected by the Heihe-Tengchong Line. **(B)** Sichuan Province's prefecture and county-level jurisdictions (Chengdu in light gray), having a similar geospatial and population density breakdown corresponding to the Heihe-Tengchong Line. **(C)** Population density of 20 county-level jurisdictions in Chengdu (data extracted from https://www.worldpop.org/geodata/summary?id=24926).

With rugged terrain and lower rainfall, West China is historically less populated and much less developed than East China (45). The Heihe-Tengchong Line bisects China from northeast to southwest, in which ~43% of Mainland China's landmass but more than 90% of its population lies southeast of the line, resulting in a population density 22 times greater than northwest of the line (46). Southwest China's subtropical Sichuan Basin is one of China's most densely populated regions, home to two of the largest urban conglomerations in the West, Chengdu and Chongqing. Sichuan Province is also the only province in China bisected by the Heihe-Tengchong Line with a similar population density/landmass bifurcation as the entire country (Figure 1). Due to its rapid urbanization, high population density, and subtropical climate (e.g., a relatively high-biomass region), this study sought to take Sichuan's capital, Chengdu, as a case study to assess the association and potential impact of three traditional measures of greenspace (e.g., NDVI, EVI, and FVC) on incidences of three major disease types (e.g., circulatory system diseases, neoplasms, and respiratory system diseases) across the city. Since many public health researchers are unfamiliar with the various greenspace indicators, we also wanted to assess the potential value of using urban ratio (e.g., % population classified as urban) as a proxy for greenspace abundance in urban health outcome studies in LMICs. We sought to answer the following research questions:

1) Does greenspace abundance have a significant association with total hospitalization rate in Chengdu?
2) Does greenspace abundance have a significant association with medical expenses paid (e.g., as an indicator of disease severity) in Chengdu (47, 48)?
3) Is there a significant difference between the three measures of greenspace abundance (e.g., NDVI, EVI, FVC) and urban ratio on the three major disease types?
4) Does landscape heterogeneity (e.g., standard deviations of greenspace indicators) have a significant association with these health outcomes?

## 2. Materials and methods

All health data utilized in this study were obtained from corresponding government agencies or derived from publicly available datasets. No human participants were involved in this study.

### 2.1. Study location

**Figure 2:**
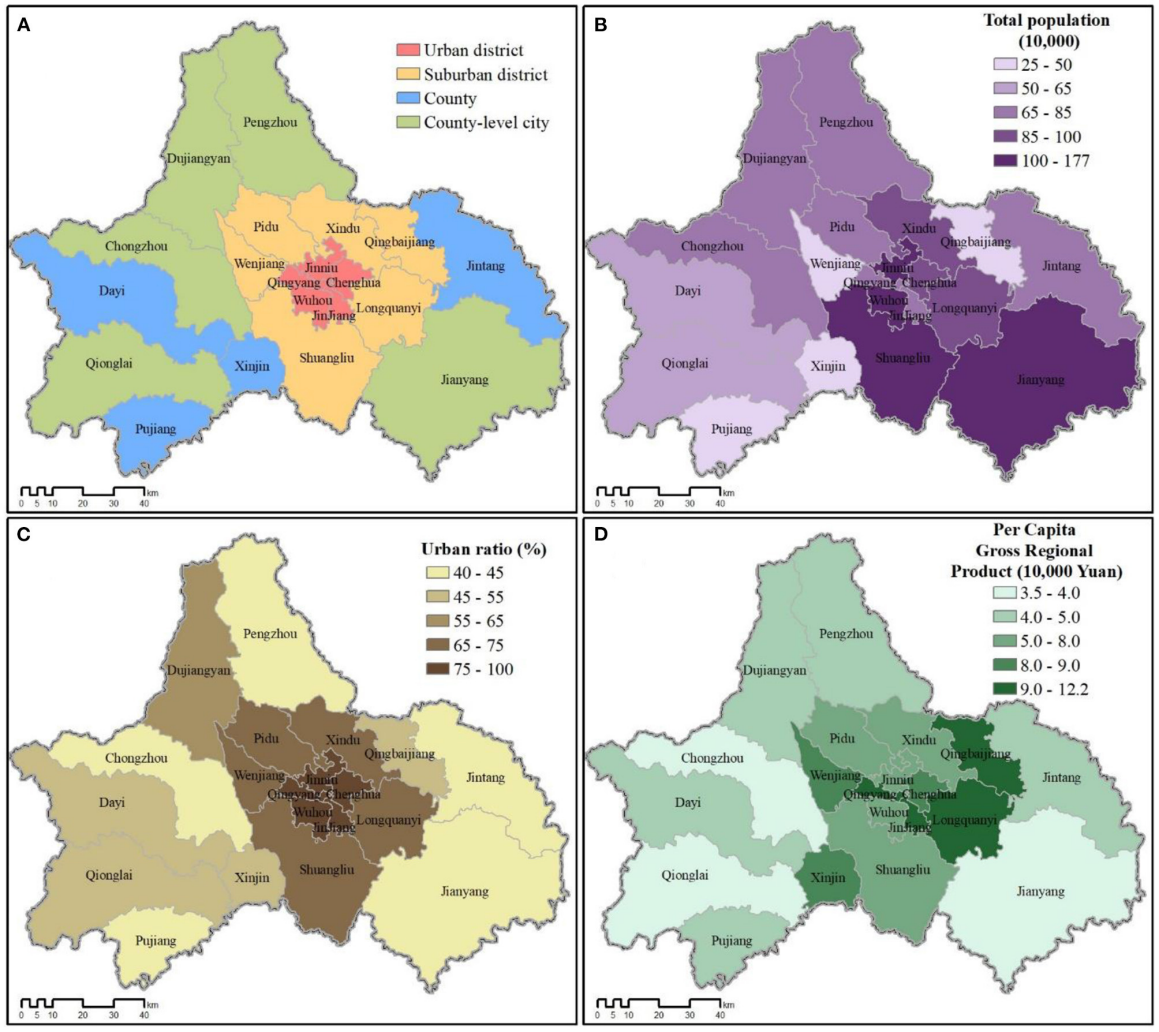
**(A)** Four types of county-level jurisdictions comprising Chengdu city. **(B)** Population by jurisdiction. **(C)** Urban ratio (% population classified as urban). **(D)** Per capital income (gross regional product; GRP) by jurisdiction. Source: Sichuan Statistical Yearbook 2017 (http://web.sctjj.cn/tjcbw/tjnj/2017/zk/indexeh.htm).

As the capital of Sichuan (92°21′~108°12′ E and 26°03′~34°19′ N) and West China's largest sub-provincial city, Chengdu (102°54′~104°53′ E and 30°05′~31°26′ N) encompasses twenty constituent county-level jurisdictions, including five urban-core districts, six rapidly-urbanizing suburban districts, four peri-urban and rural counties, and five county-level cities (Figure 2). In recent decades, Chengdu has experienced a major development boom with newly constructed residential communities and business zones expanding out across the districts and collar counties surrounding its historic urban core (49). Moreover, Chengdu's local government has strategically planned the city's green infrastructure, seeking to position itself globally as a “modern garden city” (50) or as President Jinping Xi referred to Chengdu as China's “park city.” Chengdu now has one of the highest greenspace coverage rates of any major Chinese city (36), but greenspace coverage is not uniform across its constituent jurisdictions. Thus, Chengdu is ideal for testing the impact of urban greenspace abundance on public health, representing a typical example of a rapidly-developing city in LMICs.

Data for total resident population, urban ratio (% urban), and per capita income of each jurisdiction in Chengdu (2016 calendar year end) were extracted from the Sichuan Statistical Yearbook (51). The county-level city of Jianyang (简阳市) was annexed by Chengdu in May 2016, so its data were included for all calculations and analyses.

### 2.2. Greenspace indicators

NDVI and EVI data for all constituent jurisdictions of Chengdu (2016 calendar year) were extracted from the Moderate Resolution Imaging Spectrometer (MODIS) images (h26v05 to h27v05) at the 250 m resolution, obtained from NASA's Terra MODIS product MOD13Q1 (52). Following standard practice, negative values (representing water bodies, so-called “blue-spaces”, and atmospheric obstructions) were removed before further analysis (12, 27). To calculate FVC, we utilized the following equation:


$$\begin{array}{l} FVC=\frac{\left(NDVI-NDVIsoil\right)}{\left(NDVIveg-NDVIsoil\right)} \end{array}$$

Where NDVI refers to the NDVI value of the mixed pixels, NDVI_veg_ refers to the highest NDVI value of fully-covered vegetation, and NDVI_soil_ refers to the NDVI value of bare soil (17, 18). Monthly averages, yearly averages, and standard deviations for greenspace indicators were calculated with R version 3.6.1 (53).

### 2.3. Health indicators data

Based on Sichuan's ten major causes of death in 2016 (51), we chose to analyze the impact of greenspace on the three leading disease categories (Table 1): (1) circulatory system diseases (ICD-10 I00-I99), (2) neoplasms (ICD-10 C00-D49), and (3) respiratory system diseases (ICD-10 J00-J99) (54). We acquired inpatient discharge record data for the fourth quarter of 2016 (October–December) for all twenty constituent county-level jurisdictions of Chengdu from the Health Commission of Sichuan Province (http://wsjkw.sc.gov.cn/; known before 2014 as the Health and Family Planning Commission of Sichuan Province). Every inpatient has one record in the system and indicators for healthcare institutions (e.g., institution type), basic patient demographic information (e.g., address, date-of-birth, sex, etc.), and medical process (e.g., disease code, medical fees paid) were recorded.

**Table 1:** Leading causes of death in Sichuan Province and urban areas in China (2016).

		**Sichuan Province**		**China**	
**Rank**	**Cause of death**	**Death rate (per 100,000)**	**% of Total deaths**	**Death rate (per 100,000)**	**% of Total deaths**
1	Circulatory system diseases	223.41	34.44	309.33	45.50
2	Neoplasms (tumors)	163.59	25.22	160.07	26.06
3	Respiratory system diseases	125.21	19.30	69.03	11.24
4	Trauma and toxicosis	51.50	7.94	37.34	6.08
5	Digestive system diseases	20.03	3.09	14.05	2.29
6	Endocrine, nutritional, metabolic, and immune diseases	17.20	2.55	20.43	3.33
7	Infections disease and verminosis	8.87	1.37	6.51	1.06
8	Genitourinary system diseases	7.21	1.11	6.58	1.07
9	Nervous system diseases	6.93	1.07	7.50	1.22
10	Mental disorders	2.73	0.42	2.72	0.44

Sichuan Statistical Yearbook (http://web.sctjj.cn/tjcbw/tjnj/2017/zk/indexeh.htm) and China Statistical Yearbook (http://www.stats.gov.cn/tjsj/ndsj/2017/indexch.htm).

### 2.4. Data analysis

Based on inpatient discharge records, we identified every patient's home residency jurisdiction. For each disease category, hospitalization rates (%) per total resident population were calculated for each jurisdiction, as well as average expenditure for each inpatient visit, with both used as dependent variables. We summarized monthly and yearly averages of all three greenspace indicators (NDVI, EVI, and FVC) by jurisdiction, calculating standard deviations for each, and, along with urban ratio, used these as independent variables in subsequent analyses. We then conducted Pearson correlation analysis between the independent and dependent variables using SPSS 20.0.

## 3. Results

### 3.1. Spatial variations analysis

**Figure 3:**
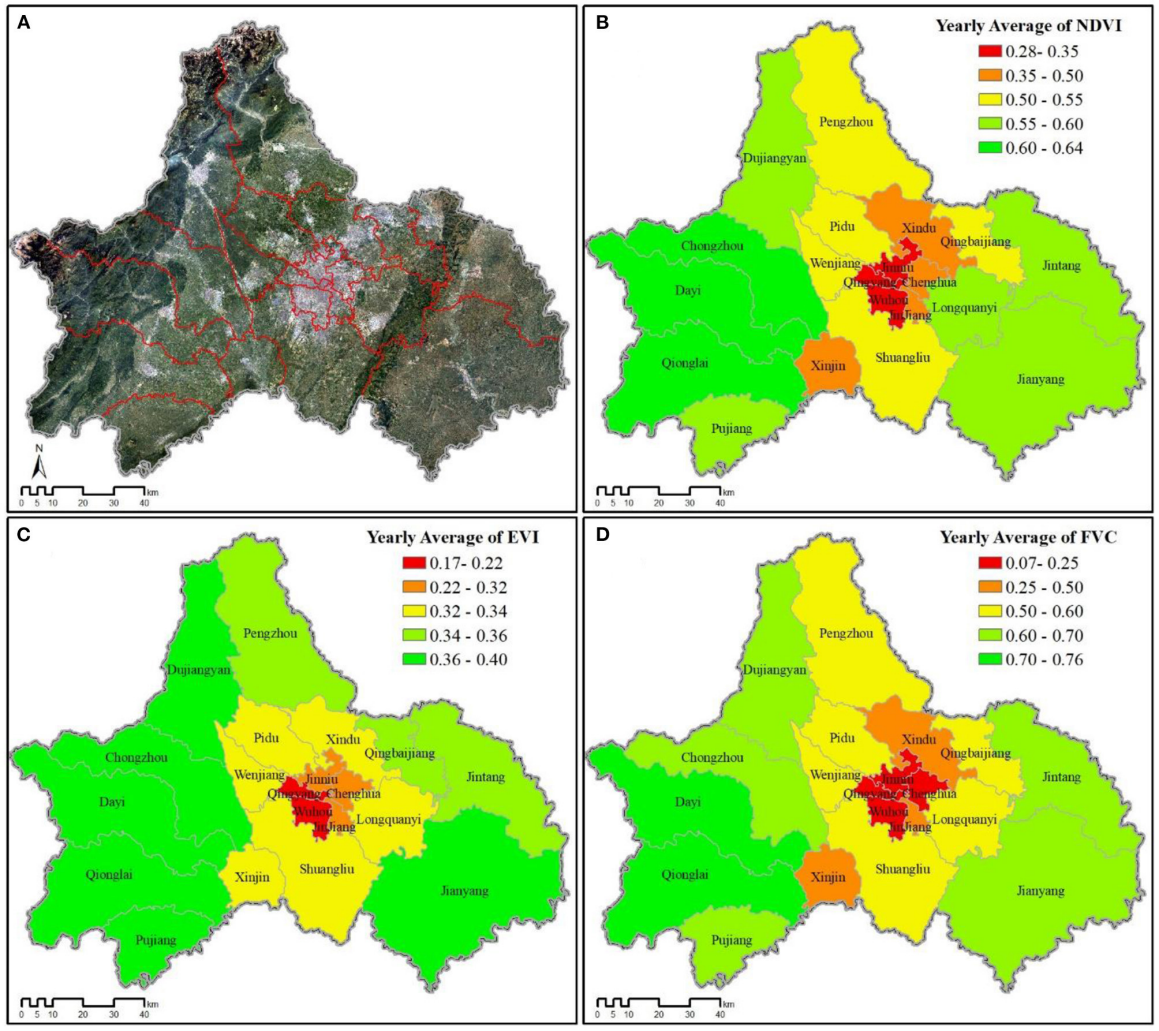
Greenspace variability for all county-level jurisdictions across Chengdu (2016). **(A)** Satellite image of Chengdu. **(B)** Yearly NDVI average. **(C)** Yearly EVI average. **(D)** Yearly FVC average.

All three greenspace abundance indicators showed similar geographic patterns across Chengdu, with the five historic urban core districts having the lowest “greenness” overall (Figure 3). However, the similar pattern shared by NDVI (Wuhou: 0.2784; Jinniu: 0.3229; Qingyang: 0.3272; Jinjiang: 0.3853; Chenghua: 0.3594; along with Xindu: 0.4576 and Xinjin: 0.4904) and FVC (Wuhou: 0.0670; Jinniu: 0.1436; Qingyang: 0.1499; Chenghua: 0.2110; Jinjiang: 0.2646; along with Xindu: 0.4050 and Xinjin: 0.4156) differed slightly from EVI (Wuhou: 0.1724; Qingyang: 0.2197; Jinniu: 0.2219; Jinjiang: 0.2265; Chenghua: 0.2279), which more clearly showed concentric bands of greater greenness moving further out from Chengdu's urban core (Figure 3C).

**Figure 4:**
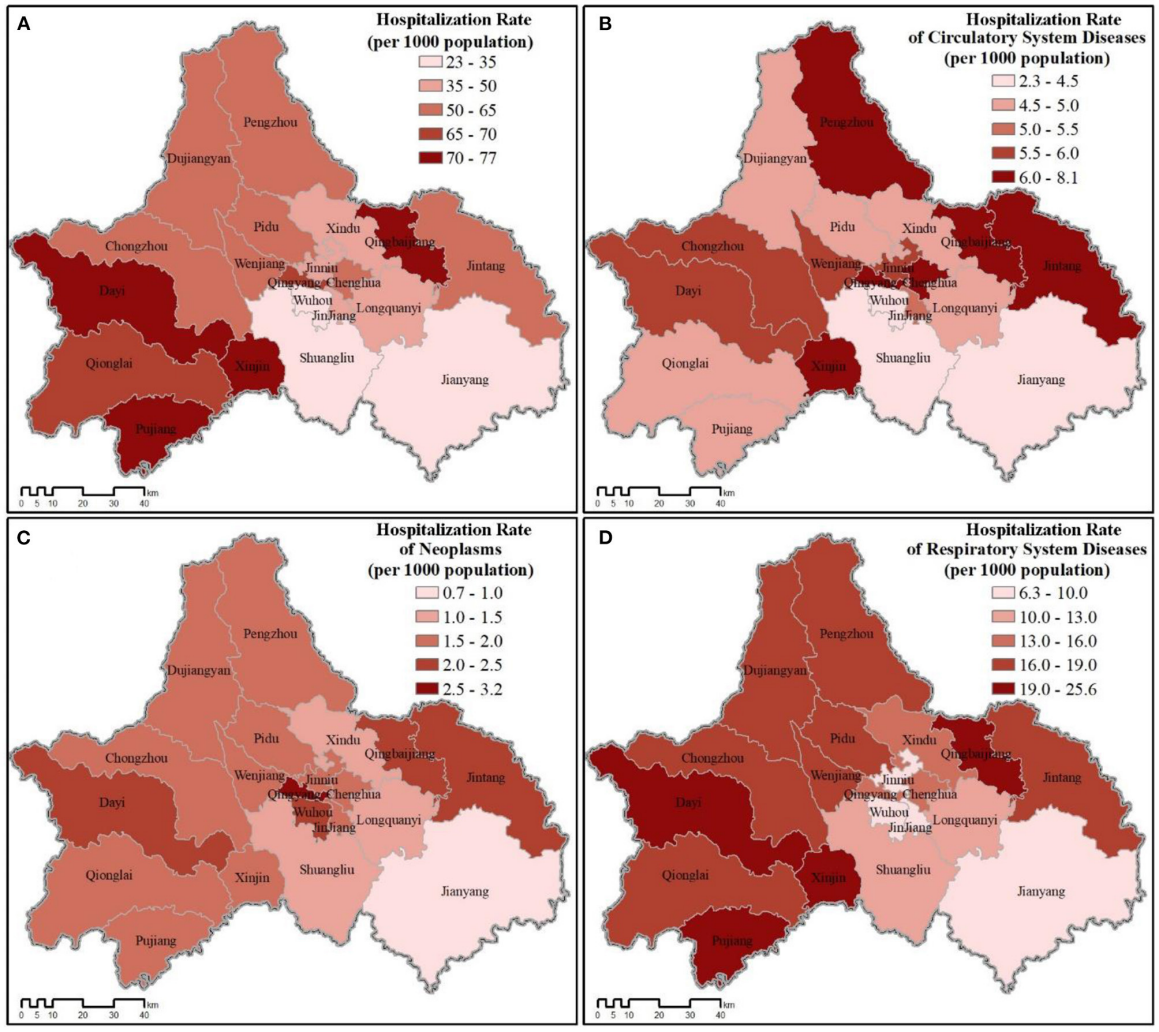
Hospitalization rate for all county-level jurisdictions across Chengdu (2016). **(A)** Total hospitalization rate. **(B–D)** Hospitalization rate for each disease category.

The total hospitalization rate varied among the twenty county-level jurisdictions, but the geographic patterns of hospitalization also differed by disease category (Figure 4). Most of the more rural jurisdictions (e.g., Xinjin: 0.0256; Dayi: 0.0237; Qingbaijiang: 0.0215; Pujiang: 0.0192; Jintang: 0.0188; Qionglai: 0.0174) had greater hospitalization rates for respiratory system diseases than the more urban districts (Wuhou: 0.0063; Jinjiang: 0.0086; Jinniu: 0.0088), but no clear pattern was evident for circulatory system diseases or neoplasms.

**Figure 5:**
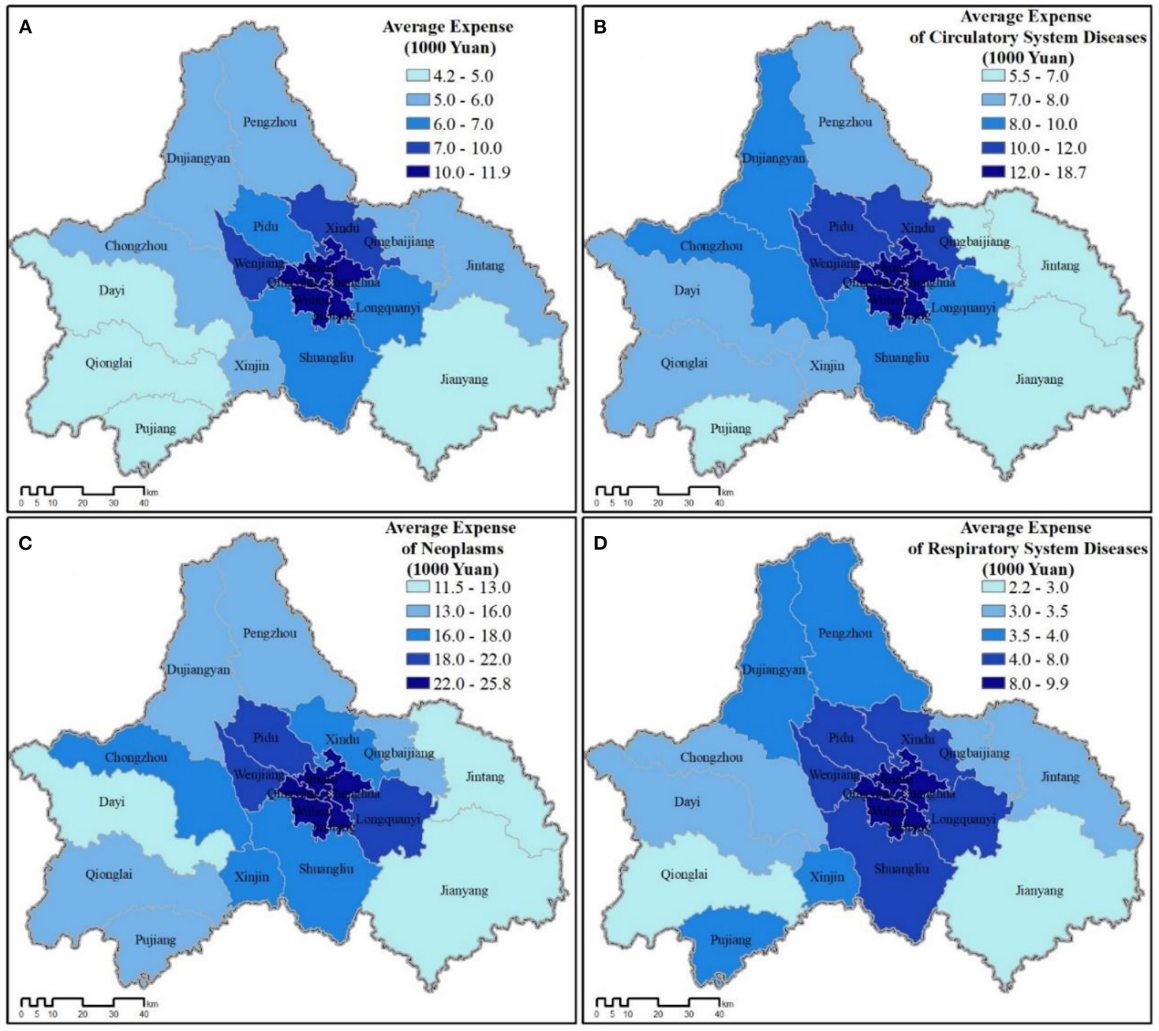
Average medical expenditures for all county-level jurisdictions across Chengdu (2016). **(A)** Total expenses. **(B–D)** Average expenses for each disease category.

The pattern of relative medical expenses paid varied across all twenty jurisdictions both for average of all expenses paid and those paid for each disease category. However, residents of the more urban districts paid more overall than the more rural jurisdictions (Figure 5). Of the three disease categories, treatment for neoplasms was the costliest, followed by circulatory system diseases and respiratory system diseases. Despite hospitalization rates tending to be greater in more rural jurisdictions for respiratory system diseases (Figure 4), residents in the more urban districts still paid more on average for treatment (Jinjiang: 9,891.92 RMB; Qingyang: 9,284.09 RMB; Jinniu: 8,765.05 RMB; Wuhou: 8,533.41 RMB; Chenghua: 8,287.14 RMB; vs. Qionglai: 2,934.15 RMB; Dayi: 3,026.47 RMB; Jintang: 3,311.87 RMB; Qingbaijiang: 3,473.43 RMB; Xinjin: 3,574.44 RMB; Pujiang: 3,695.89 RMB).

**Figure 6:**
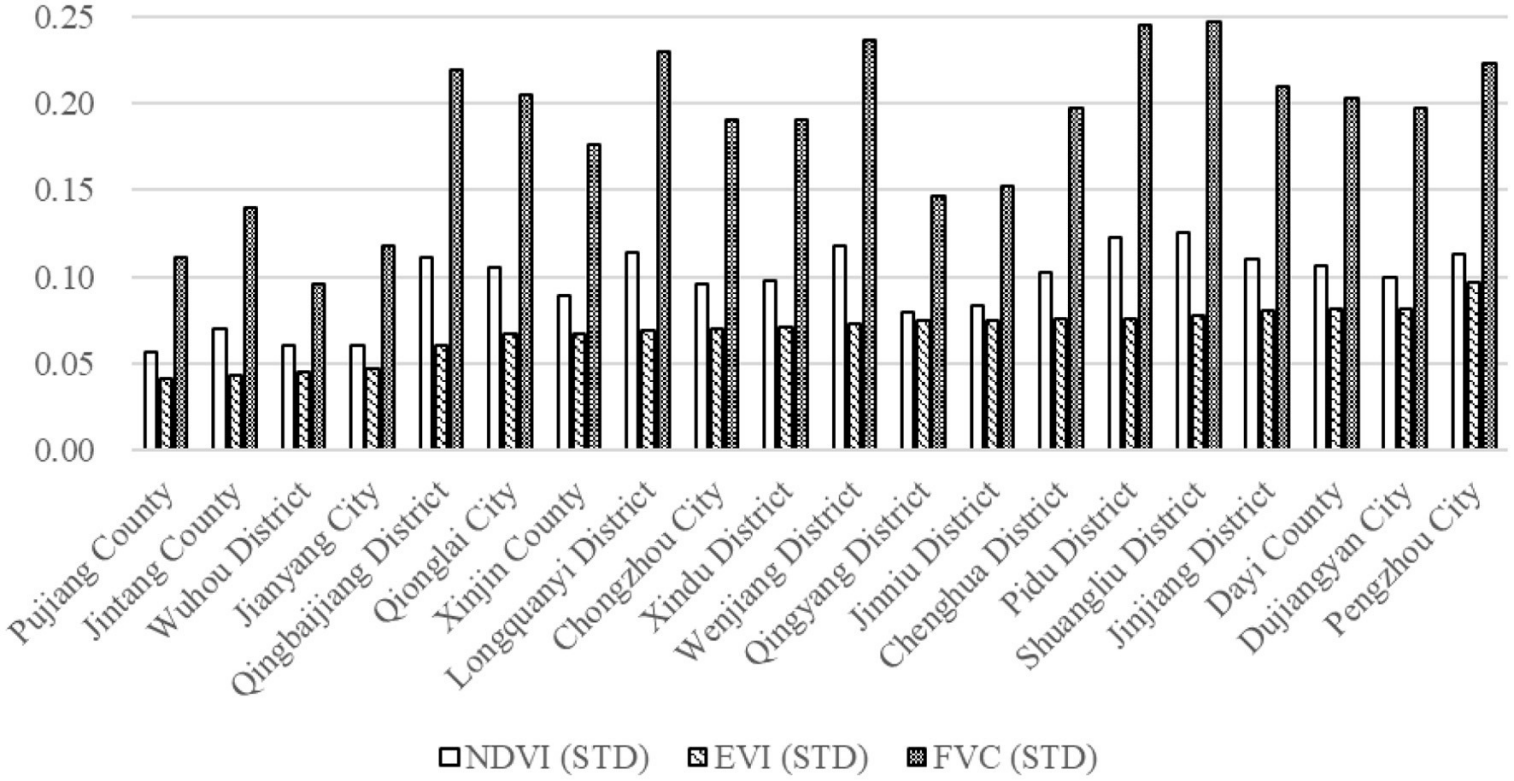
Standard deviation of greenspace indicators (2016 Yearly Average) across Chengdu's 20 constituent jurisdictions (ranked by EVI).

In terms of greenspace variability (greenspace standard deviation), FVC represented greater variability overall, but followed a similar pattern with NDVI, with EVI fluctuating the least (Figure 6).

### 3.2. Correlation analysis

All three greenspace indicators displayed significant, positive correlation with hospitalization rate for respiratory system diseases, but EVI more strongly correlated (*r* = 0.628, *p* = 0.01) than either NDVI (*r* = 0.560, *p* = 0.05) or FVC (*r* = 0.555, *p* = 0.05) (Table 2). Though not significant, all greenspace indicators negatively associated with hospitalization for circulatory system diseases and neoplasms. Standard deviations of each greenspace indicator (annual average) were not significantly associated with total hospitalization rate or hospitalization rates for any disease category (Table 2).

**Table 2:** Correlation analysis of greenspace indicators, urban ratio, and per capita GRP with hospitalization rate and average inpatient medical expenses.

		**Urban ratio**	**Per**	**Annual average**			**Standard deviation**		
			**capita GRP**	**NDVI**	**EVI**	**FVC**	**NDVI**	**EVI**	**FVC**
Urban ratio	–	0.707^**^	−0.918^**^	−0.942^**^	−0.919^**^	0.037	0.207	−0.058
Hospitalization rate	Total	−0.425	−0.175	0.337	0.403	0.333	0.078	0.071	0.117
	Circulatory System Diseases	0.092	0.246	−0.167	−0.101	−0.176	0.202	0.268	0.210
	Neoplasms	0.272	0.226	−0.370	−0.344	−0.370	−0.082	0.109	−0.128
	Respiratory System Diseases	−0.644^**^	−0.334	0.560*	0.628^**^	0.555*	0.140	0.043	0.203
Average inpatient medical expenses	Total	0.966^**^	0.635^**^	−0.930^**^	−0.954^**^	−0.930^**^	−0.069	0.192	−0.167
	Circulatory system diseases	0.930^**^	0.615^**^	−0.861^**^	−0.877^**^	−0.862^**^	−0.010	0.258	−0.102
	Neoplasms	0.947^**^	0.654^**^	−0.872^**^	−0.891^**^	−0.873^**^	0.028	0.222	−0.054
	Respiratory system diseases	0.947^**^	0.648^**^	−0.920^**^	−0.948^**^	−0.921^**^	−0.101	0.174	−0.197

Significance level: ^*^p < 0.05 and ^**^p < 0.01.

Urban ratio exhibited significant negative association with monthly (Table 3) and yearly averages of all three greenspace indicators (Table 2), meaning that higher urban ratios have lower overall greenness. This strong association was robust against seasonal fluctuations among greenspace indicators. Furthermore, urban ratio (e.g., less greenness) was significantly associated (negatively) with hospitalization for respiratory system diseases (*r* = −0.644, *p* = 0.01), indicating higher urban ratios associated with lower hospitalization rates for respiratory system diseases (Table 2). Though not significant, urban ratio also negatively associated with total hospitalization but positively associated with hospitalization rates for circulatory system diseases and neoplasms.

**Table 3:** Correlation between greenspace indicator monthly averages and urban ratio (2016).

	**NDVI**	**EVI**	**FVC**
January	−0.917^**^	−0.902^**^	−0.919^**^
February	−0.931^**^	−0.923^**^	−0.932^**^
March	−0.900^**^	−0.882^**^	−0.905^**^
April	−0.879^**^	−0.875^**^	−0.881^**^
May	−0.840^**^	−0.827^**^	−0.838^**^
June	−0.888^**^	−0.903^**^	−0.888^**^
July	−0.927^**^	−0.926^**^	−0.927^**^
August	−0.871^**^	−0.896^**^	−0.874^**^
September	−0.863^**^	−0.896^**^	−0.864^**^
October	−0.852^**^	−0.879^**^	−0.852^**^
November	−0.878^**^	−0.876^**^	−0.878^**^
December	−0.923^**^	−0.952^**^	−0.922^**^

Significance level: ^**^p < 0.01.

Urban ratio displayed significant positive correlation with relative wealth (per capita GRP; *r* = 0.707, *p* = 0.01). Relative wealth also significantly associated (negatively) with all three greenspace indicators, including NDVI (*r* = −0.645, *p* = 0.01), EVI (*r* = −0.657, *p* = 0.01), and FVC (*r* = −0.649, *p* = 0.01). However, relative wealth was not significantly associated with any hospitalization rate, although associations with total hospitalization and hospitalization for respiratory system diseases were negative, while associations between wealth and hospitalization rates for the other two disease categories were positive.

**Figure 7:**
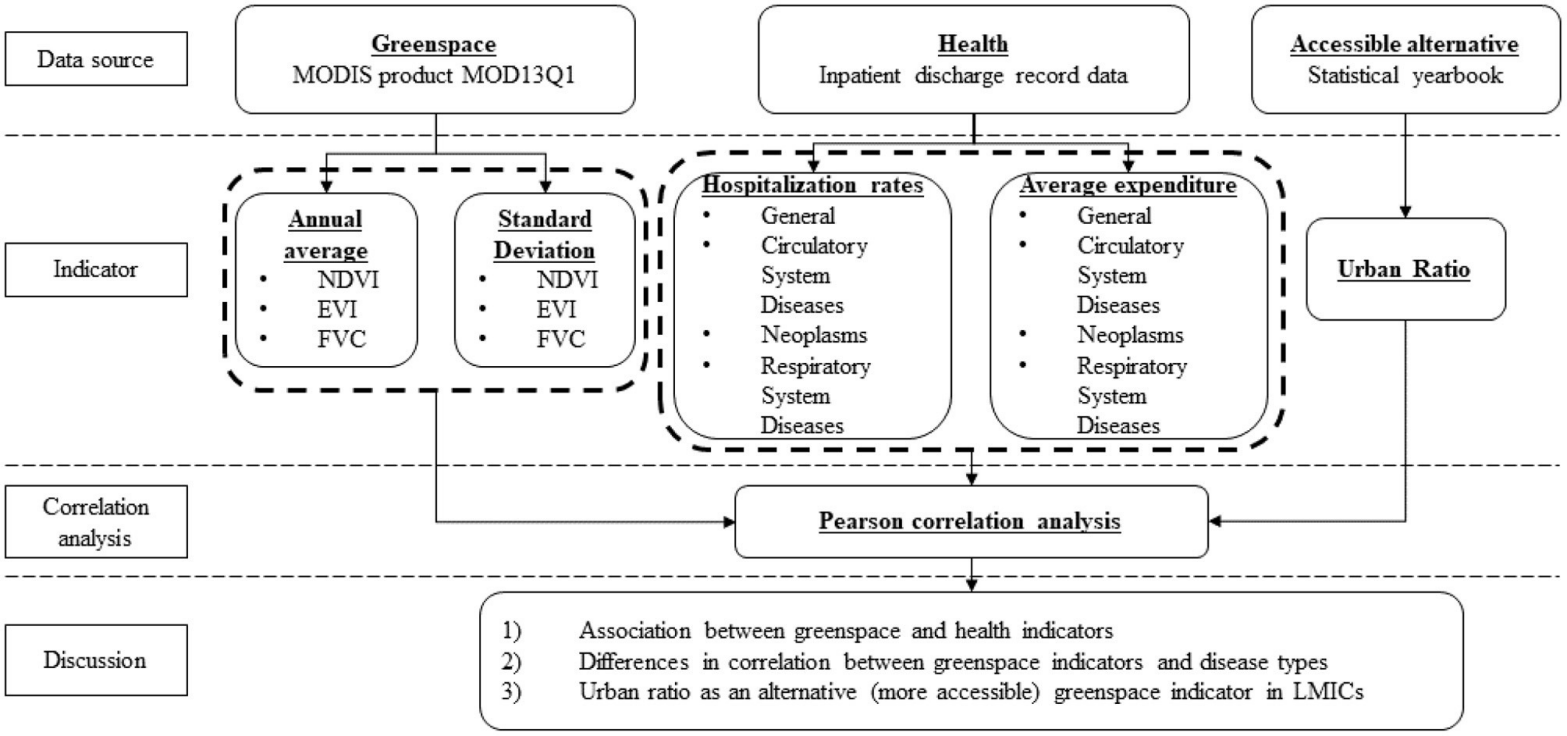
Flow chart of research and analysis.

Urban ratio demonstrated significant positive association with average inpatient medical expenses, indicating that higher urban ratios were associated with higher medical expenditures per visit (Table 2). This significant association held true for total expenditures (*r* = 0.966, *p* = 0.01) and all three disease categories (*p* = 0.01). Relative wealth also significantly associated (positively) with total medical expenses (*r* = 0.635, *p* = 0.01) and medical expenses paid for all three disease categories (*p* = 0.01). All three greenspace indicators strongly associated (negatively) with inpatient medical expenses paid (Table 2), in total and for expenses paid for each disease category (*p* = 0.01). However, of the three indicators, annual average EVI had the highest correlation with all expense categories (Table 2). The association between the greenness indicator standard deviations and inpatient expenditures were insignificant (Table 2). A flowchart of our research methods and analyses is shown in Figure 7.

## 4. Discussion

Urbanization not only disconnects people from nature access traditionally enjoyed by rural communities (29, 55, 56), modern urban lifestyles have further reduced nature access through increased computer, television, and mobile screen time (7). Thus, accessibility of “walkable” greenspaces nearby residential communities has a direct positive impact on physical health by reducing sedentary behaviors of vulnerable urban populations including the elderly (6) and youths (5). Studies have also documented a positive association between public greenspace access and greater psychological health (21, 22, 57), social cohesion (58–60), as well as cognitive attention and educational attainment (61, 62). Other indirect benefits of urban greenspaces on public health include intercepting and reducing air (39, 63) and noise pollution (28, 64), as well as moderating ambient temperatures leading to more comfortable urban lifestyles (40, 41, 65).

Reviewing 125 greenspace studies, Taylor and Hochuli (9) found that less than half of the papers clearly defined what the researchers meant by “greenspace.” Most that did provide a definition fell within two broad characterizations: (1) an overarching concept of nature in which greenspace is essentially a “synonym of nature and antonym of urbanization,” or (2) urban vegetation itself, including various types of vegetated spaces found in urban environments (9). Our findings demonstrate how widely different implications can be drawn depending on which of these definitions is used to assess the impact of greenspace abundance on human health and wellbeing.

### 4.1. Greenspace abundance and total hospitalization

Contrary to expectations, annual averages of the three greenspace abundance indicators did not significantly correlate with total hospitalization rate in Chengdu. This may be explained by confounding variables due to seasonality and plant phenology. For example, during times of seasonal transitions, other factors may impact health outcomes in terms of plant pollen, fungal spores, and temperature changes, all of which can have rapid impacts on human health that differ by disease category. The lack of significant association between greenspace abundance and total hospitalization may also be partially explained by epidemiological confounding variables, such as age, sex, and smoking history, as well as by temporal confounding variables of hospitalization such as day-of-the-week and holiday effects. Thus, more research is necessary to determine what seasonal variables may interact with greenspace abundance in Chengdu to predict human health impacts.

### 4.2. Greenspace abundance and medical expenses paid

As expected, greenspace abundance does significantly associate with medical expenses paid, in that all three measures of greenspace significantly correlated (negatively) with all categories of average inpatient medical expenses paid during the 2016 calendar year (Table 2). We originally wanted to test whether medical expenses paid could be used as a proxy for disease severity, assuming more severe cases would require greater treatment expenses (47, 48). Yet, we began to suspect in Chengdu average expenses paid was more likely representative of relative wealth than disease severity. Although relative wealth was not significantly associated with any hospitalization rate, it was significantly associated (positively) with total medical expenses (*r* = 0.635, *p* = 0.01), medical expenses for all disease categories (*r* = 0.615–0.654, *p* = 0.01), and with urban ratio (*r* = 0.707, *p* = 0.01). Conversely, relative wealth negatively associated with all three greenspace indicators, implying that within greater Chengdu, urban areas with less greenspace tend to be wealthier (Figures 2C, 3).

Though hospitalization rates for respiratory system diseases were greater in less urban jurisdictions, residents in urban districts still paid more on average for treatment. This shows that our initial assumption that “cost” was a proxy for disease severity was inaccurate. In China, there are three tiers of healthcare institutions (66, 67), based on the responsibilities and healthcare services provided (defined by the government). Besides tiers, there are also three levels of healthcare institutions, based on service capacity (e.g., bed space, number of doctors, equipment, etc.,). Most higher-category hospitals are both third tier and third level, but not all first level hospitals are first tier. Within this healthcare institution categorization scheme, the price charged for a particular treatment is the same across all healthcare institutions in the same tier regardless of where they are located, but prices increase at each successive tier. For more serious conditions, patients often choose to get treatment at the highest-tier institution they can afford, whereas they tend toward lower-tier institutions (community health clinics) for more common ailments. It is important to note, however, that rural people frequently travel to higher tier medical institutions in urban centers when they have severe diseases, but, in our study, we used place of *residence* not place of *treatment* for our analyses. Thus, we found relative wealth likely determined why people paid more for certain diseases (e.g., they tended to choose the highest-tier hospitals within their budget), rather than disease severity. Consequently, teasing out the impact of greenspace on this variable proved difficult due to the confounding correlation of relative wealth.

### 4.3. Association of greenspace indicators on health outcomes

The impact of NDVI on hospitalization rates for circulatory diseases and neoplasms was not significant, but there was a significant association for respiratory disease, indicating greenspace abundance can indeed have significant association with certain categories of disease (Table 2). Moreover, we found different patterns between the three greenspace indicators. All three greenspace indicators significantly correlated (positively) with hospitalization rates for respiratory diseases, but EVI correlated much more strongly (Table 2). EVI not only had an overall higher correlation with significant health outcomes, but its associational pattern also fluctuated less than the other two greenspace indicators (Figure 6, Table 3). This may be due to the fact that unlike NDVI (and by extension FVC), EVI avoids reflection distortions and does not become saturated in high-density vegetation (14, 15). Thus, of the three traditional measures of greenness, we found EVI to be the best overall.

We also wanted to identify whether the three greenspace indicators and urban ratio correlated or differed significantly in their overall association with each health outcome. We found that urban ratio was significantly (negatively) associated with the monthly and yearly averages of all three greenspace indicators (Tables 2, 3). Urban ratio seemed to function nearly opposite to EVI (*r* = 0.628, *p* = 0.01) in its association with hospitalization for respiratory system diseases (*r* = −0.644, *p* = 0.01). For public health researchers who may find traditional greenspace indicators difficult to calculate without having geographic research backgrounds, we found urban ratio to be an easier to calculate and acceptable proxy for greenness (opposite EVI). Essentially, higher urban ratios mean lower overall greenness. Thus, for convenience, in LMICs where urban ratio is more likely to imply less greenness, urban ratio would be an acceptable negative indicator of greenness, especially in studies utilizing Taylor and Hochuli's (9) first definition of greenspace as being a “synonym of nature and antonym of urbanization.”

Nevertheless, despite finding significant correlation between greenspace abundance and certain health outcomes, our results were counter intuitive. Contrary to expectations for a negative correlation between greenspace abundance and hospitalization rate (which was true for circulatory diseases and neoplasms, though not significantly), we found a significant positive correlation between all indicators of greenspace abundance and respiratory disease hospitalization (as with total hospitalization, though not significant). Thus, as Chengdu's greenspace abundance goes up (and urban ratio goes down), the hospitalization rate for respiratory disease increases (Table 2). It is important to note that both circulatory disease and neoplasms may experience more delayed effects from greenspace abundance, whereas respiratory disease is more immediate (with seasonal variations). Thus, as the impact of greenspace on human health is complicated and difficult to tease apart from other variables, with both direct (e.g., removal of air pollutants) and indirect (e.g., more physical activities and better mood) impacts (68), further exacerbated by the spatial lag effect, more population-level studies are needed.

Moreover, under further scrutiny, the unexpected associations we found between greenspace abundance and human health may be due to differing demographic characteristics and lifestyle patterns of the various county-level jurisdictions within Chengdu. For example, in the more rural county-level jurisdictions in the north and west of Chengdu where hospitalization rates for respiratory disease are higher, there are greater retirement communities and assisted-care facilities in these jurisdictions known for natural forests, relatively clean air, and abundant green areas (e.g., Chongzhou, Dujiangyan, and Pengzhou cities, as well as Dayi County). This may be one reason to explain why city-wide investigations of greenspace abundance on human health outcomes may differ significantly from studies at other scales (26). Areas with greater “greenness” may self-select over-time for older populations with greater incidences of certain diseases than the wider population, or greater greenness may otherwise encourage sedentary behaviors. Consequently, further studies are also necessary to tease out potentially confounding variables at various scales when investigating the direct effect of greenspace abundance on human health.

### 4.4. Landscape heterogeneity and health outcomes

Based on the findings of Pereira et al. (27), we wanted to test whether landscape heterogeneity (e.g., standard deviations of the greenspace indicators) would have a significant association with human health outcomes. In terms of greenspace variability, FVC represented greater variability overall, but followed a similar pattern with NDVI, but EVI fluctuated the least. Since we found EVI to be the best overall measure of greenness in Chengdu, when ranking Chengdu's 20 jurisdictions by EVI standard variation (Figure 6), assessing the top five and bottom five most variable jurisdictions yields interesting insights. Of the five least variable jurisdictions, Pujiang County, Jintang County, and Jianyang City are all largely rural with large amounts of farmland. Wuhou District (the third least variable jurisdiction) is one of the most heavily urbanized districts in Chengdu's urban core, while Qingbaijiang District is a moderately urbanized suburban district. All five of these least-variable jurisdictions represent different levels of urbanization and greenness, but landscape characteristics are rather uniform across their respective jurisdictions.

In contrast, the five most variable county-level jurisdictions include Pengzhou City, Dujiangyan City, and Dayi County, all of which have exurban enclaves nestled among rolling mountains and forests, indicating greater contrast in their respective landscapes. Jinjiang District (the 4th most variable jurisdiction) is also a heavily urbanized district, but unlike Wuhou with an older, more uniform urban landscape, Jinjiang includes large parks and riparian greenspaces as well as many recently redeveloped residential communities enclosing lush landscapes of maintained greenspaces. These landscape features add to the overall greenspace variability in Jinjiang District. Finally, Shuangliu District is a rapidly urbanizing suburban district that still has extensive tracts of farmland interspersed with newly constructed residential communities and business zones, as well as the international airport, thereby comprising one of the most variable landscapes.

Thus, depending on which definition of greenspace is employed, different county-level jurisdictions would be favored in subsequent analyses. In Chengdu, the northern and western jurisdictions are mountainous and tend to be less evenly developed, with large tracts of forested areas (though they still have relatively high human population densities). Moreover, most suburban collar-districts, counties, and county-level cities have extensive farmland. Thus, these jurisdictions would likely be favored in studies utilizing the first definition of greenspace as being synonymous with nature/antonymous with urbanization (9). Yet, despite these jurisdictions having greater “greenness” overall, the large number of retirement communities, second homes, and elder-care facilities in these jurisdictions may confound the benefit of greenness due to more sedentary, older populations.

However, in the central urban core districts, the second type of greenspace identified by Taylor and Hochuli (9), that of urban vegetation, becomes more important. Comparing the landscape variability of Jinjiang and Wuhou districts, despite similar levels of urbanization (e.g., urban ratio; Figure 2), Jinjiang has a greater proportion of greenspace by all three indicators (NDVI: 0.3854; EVI: 0.2265; FVC: 0.2646) vs. Wuhou (NDVI: 0.2784; EVI: 0.1724; FVC: 0.0670; Figure 3). Yet, Takano et al. (6) noted the importance of not just quantity and quality of greenspace, but also *access* to the greenspace. For example, a recent study in China's largest city, Shanghai found that the city's outer suburban county-level jurisdictions lagged behind the city as a whole in improving greenspace accessibility (69). Thus, in some ways, Chengdu's more rural jurisdictions despite having greater abundance of greenspace may have more difficulty accessing it, with elderly residents less able to mountain climb. In contrast, the more urban districts with greenspace located within short walking distance of residential communities may actually encourage greater use of their less abundant greenspaces.

Nevertheless, recent studies in Chengdu have shown that even when greenspace is accessible, it does not necessarily mean it is utilized (56, 70). For example, one recent study found that although urban greenspace coverage across the urban core of Chengdu had reached 37.71% (2018), only 27.49% of this greenspace was used efficiently (70). Similarly, another study recently found that despite Chengdu's extensive investment in building greenspace infrastructure across the city, these spaces may be largely serving only as “backdrops” to urban living, rather than being fully-utilized with active and passive educational opportunities (56). Thus, more research is necessary to further tease out the implications of landscape heterogeneity on human health and the various confounding variables, including the differences between public perceptions of greenspace quality and quantity, as well as theoretical accessibility vs. actual utilization, on multiple scales.

### 4.5. Limitations and future direction

In epidemiological research, how greenspace is measured (both in terms of quantity and quality) has been the subject of much recent debate (10, 12, 13, 23, 36). For example, although NDVI is frequently used as a metric to quantify greenspace exposure (11), as we found, when various greenspace metrics are compared, their association with health outcomes can differ significantly (12, 13, 23). In addition, considering the two general definitions of greenspace identified by Taylor and Hochuli (9), Akpinar et al. (8) found significant impact of greenspace on human physical and mental health when comparing various definitions of greenspace, including forested areas and urban vegetation, but there was no significant affect when these different types of greenspace were aggregated together. Due to how different kinds of greenspace are utilized, spatial variability and accessibility may be more important than absolute abundance of greenspace in terms of promoting active lifestyles (27). Thus, future studies must clearly define what is meant by greenspace and justify how it is quantified at each scale of analysis.

Furthermore, de Vries et al. (60) found that greenspace *quality* not just *quantity* had significant effects on human health and wellbeing. Qualitative aspects of greenspace (e.g., type of vegetation, biodiversity, greenspace functions and amenities, etc.) influence how it is perceived and utilized by the local community, but these aspects are infrequently taken into consideration in health outcome studies (10–13). Moreover, throughout the year, people have different interactions with greenspace, generally having more physical activities in spring and autumn, but fewer in winter. In addition, diverse tree species in greenspaces provide different ecosystem functions and services, for example, deciduous trees can provide thermal cooling effects during summer, while evergreen trees provide wind protection in winter. This may help to explain why we found no significant effect of landscape variability (e.g., greenspace standard deviation) on health outcomes in this study, since we did not specifically assess how local people were using or perceiving these spaces.

We identified many epi-confounding factors that could potentially explain the relationship between greenspace and city-wide population-level health outcomes. However, in this study we could only access some of the data to test these questions. That is, due to data limitations, as well as logistical considerations for this single research study, only a simple correlation analysis was carried out to assess the potential relationship between greenspace abundance indicators and health outcomes. For example, Chengdu does not report average age of each constituent county-level jurisdiction, and lifestyle patterns and retirement communities likely further confound this variable. In addition, seasonality and plant phenology patterns also require more research, since greenspace indicators can fluctuate seasonally along with temperature, humidity, and plant phenology (13, 15, 23). Moreover, we found significant effects of greenspace abundance on respiratory disease hospitalization, so understanding how spatial variability of air quality affects respiratory disease also requires further research (71). As vehicular traffic increases, anthropogenic volatile organic compounds (VOCs) combine with plant-based biogenic VOCs as precursors to ground-level ozone, which damages the human respiratory system (42, 72–74). Since vegetation composition differs between heavily urbanized and forested, less-urban areas, not understanding how much biogenic VOCs are produced by different types of vegetation or how it affects human health may weaken or otherwise bias results (73, 74). Thus, our findings suggest that more methods should be developed and utilized to further disentangle the complex effect of greenspace on human health. In particular, teasing out the positive and negative impacts of urban vegetation becomes increasingly important.

## 5. Conclusion

The main purpose of this research was to investigate the selection of appropriate greenspace indicators in studies (like this one) with limited data availability and verify that the effect of greenspace differs on different disease types. Overall, EVI was the best measure of greenspace abundance in Chengdu. We found greenspace does have a significant impact on public health across the city, but this relationship differed by disease type, with greenspace having significant positive association with respiratory disease, but insignificant negative associations with the other disease categories. We also found urban ratio significantly (negatively) associated with greenspace abundance, meaning that jurisdictions with higher urban ratios have less greenness. Moreover, the higher the urban ratio (e.g., less greenspace), the more medical expenses are paid. This relationship was found in terms of both the urban ratio, which positively correlated with medical expenses, as well as all three greenspace indicators, which negatively correlated with medical expenses. Consequently, in future health outcome studies, urban ratio would be an acceptable negative indicator of greenness in LMICs where urban ratio likely implies less greenness. Future studies should articulate clear definitions of greenspace and justify how it is quantified at each scale of analysis. Assessments of landscape variability (in terms of how greenspace quantity is mitigated by quality) should also be incorporated in future study designs. In addition, future health outcome studies should assess how local people perceive (quality) and utilize (access) greenspaces to better tease out confounding variables on health outcomes and provide better guidance for local government policies and greenspace design.

## Data availability statement

The data analyzed in this study is subject to the following licenses/restrictions: Greenspace abundance indicators utilized in the manuscript were calculated from public data, while individual inpatient records were utilized to calculate human wellbeing indicators, and the latter data are not publicly available. Requests to access these datasets should be directed to wang_xiuli@scu.edu.cn.

## Author contributions

BCS and XW planned and designed the research. BCS, HL, XW, YX, and YW collected and analyzed the data. BCS, HL, XW, and SZ interpreted the data. BCS wrote the manuscript. HL, XW, and SZ revised and provided feedback to critically improve the manuscript. All authors made significant contributions to complete this work.

## References

[1] UNDESA. World Urbanization Prospects: The 2018 Revision. New York (2019).

[2] Ekkel ED de Vries S. Nearby green space and human health: Evaluating accessibility metrics. Landsc Urban Plan. (2017) 157:214–20. 10.1016/j.landurbplan.2016.06.008

[3] Bertram C Rehdanz K. The role of urban green space for human well-being. Ecol Econ. (2015) 120:139–52. 10.1016/j.ecolecon.2015.10.013

[4] Shuvo FK Feng X Akaraci S Astell-Burt T. Urban green space and health in low and middle-income countries: a critical review. Urban Urban Green. (2020) 13:126662. 10.1016/j.ufug.2020.126662

[5] Epstein LH Raja S Gold SS Paluch RA Pak Y Roemmich JN. Reducing sedentary behavior: the relationship between park area and the physical activity of youth. Psychol Sci. (2006) 17:654–659. 10.1111/j.1467-9280.2006.01761.x 16913945

[6] Takano T Nakamura K Watanabe M. Urban residential environments and senior citizens' longevity in megacity areas: the importance of walkable green spaces. J Epidemiol Community Heal. (2002) 56:913–8. 10.1136/jech.56.12.913 12461111 PMC1756988

[7] Frumkin H Bratman GN Breslow SJ Cochran B Kahn PH Lawler JJ. Nature contact and human health: a research agenda. Environ Health Perspect. (2017) 125:1–18. 10.1289/EHP1663 28796634 PMC5744722

[8] Akpinar A Barbosa-leiker C Brooks KR. Does green space matter? Exploring relationships between green space type and health indicators. Urban For Urban Green. (2016) 20:407–418. 10.1016/j.ufug.2016.10.013

[9] Taylor L Hochuli DF. Defining greenspace: multiple uses across multiple disciplines. Landsc Urban Plan. (2017) 158:25–38. 10.1016/j.landurbplan.2016.09.024

[10] Dzhambov AM Browning MHEM Markevych I Hartig T Lercher P. Analytical approaches to testing pathways linking greenspace to health: a scoping review of the empirical literature. Environ Res. (2020) 186:109613. 10.1016/j.envres.2020.109613 32668553

[11] James P Banay RF Hart JE Laden F. A review of the health benefits of greenness. Curr Epidemiol Rep. (2015) 2:131–42. 10.1007/s40471-015-0043-7 26185745 PMC4500194

[12] Markevych I Schoierer J Hartig T Chudnovsky A Hystad P Dzhambov AM. Exploring pathways linking greenspace to health: Theoretical and methodological guidance. Environ Res. (2017) 158:301–17. 10.1016/j.envres.2017.06.028 28672128

[13] Rugel EJ Henderson SB Carpiano RM Brauer M. Beyond the normalized difference vegetation index (NDVI): developing a natural space index for population-level health research. Environ Res. (2017) 159:474–83. 10.1016/j.envres.2017.08.033 28863302

[14] Huete A Didan K Miura T Rodriguez EP Gao X Ferreira LG. Overview of the radiometric and biophysical performance of the MODIS vegetation indices. Remote Sens Environ. (2002) 83:195–213. 10.1016/S0034-4257(02)00096-2

[15] Fensholt R Sandholt I Stisen S. Evaluating MODIS, MERIS, and VEGETATION vegetation indices using in situ measurements in a semiarid environment. IEEE Trans Geosci Remote Sens. (2006) 44:1774–86. 10.1109/TGRS.2006.875940

[16] Carlson TN Ripley DA. On the relation between NDVI, fractional vegetation cover, and leaf area index. Remote Sens Environ. (1997) 62:241–52. 10.1016/S0034-4257(97)00104-127879814

[17] Liang S Wang J. Fractional Vegetation Cover. Advanced Remote Sensing: Terrestrial Information Extraction and Applications. Cambridge, MA: Academic Press (2020). p. 477–510. 10.1016/B978-0-12-815826-5.00012-X

[18] Li M Wu B Yan C Zhou W. Estimation of vegetation fraction in the upper basin of miyun reservoir by remote sensing. Resour Sci. (2004) 26:153–9. Available online at: https://www.cnki.com.cn/Article/CJFDTOTAL-ZRZY200404022.htm

[19] Chu D. Remote Sensing of Land Use and Land Cover in Mountain Region. (2020). 10.1007/978-981-13-7580-431161233

[20] de Vries S Verheij RA Groenewegen PP Spreeuwenberg P. Natural environments—Healthy environments? An exploratory analysis of the relationship between greenspace and health. Environ Plan A. (2003) 35:1717–31. 10.1068/a3511135954952

[21] Wood L Hooper P Foster S Bull F. Public green spaces and positive mental health—investigating the relationship between access, quantity and types of parks and mental wellbeing. Health Place. (2017) 48:63–71. 10.1016/j.healthplace.2017.09.002 28942343

[22] Van Den Berg M Van Poppel M Van Kamp I Andrusaityte S Balseviciene B Cirach M. Visiting green space is associated with mental health and vitality: a cross-sectional study in four european cities. Health Place. (2016) 38:8–15. 10.1016/j.healthplace.2016.01.003 26796323

[23] Larkin A Hystad P. Evaluating street view exposure measures of visible green space for health research. J Expo Sci Environ Epidemiol. (2018) 29:1–10. 10.1038/s41370-018-0017-1 29352209

[24] Markevych I Feng X Astell-Burt T Standl M Sugiri D Schikowski T. Residential and school greenspace and academic performance: evidence from the GINIplus and LISA longitudinal studies of German adolescents. Environ Pollut. (2019) 245:71–6. 10.1016/j.envpol.2018.10.053 30414551

[25] Gascon M Mas MT Martínez D Dadvand P Forns J Plasència A. Mental health benefits of long-term exposure to residential green and blue spaces: a systematic review. Int J Environ Res Public Health. (2015) 12:4354–79. 10.3390/ijerph120404354 25913182 PMC4410252

[26] Richardson EA Mitchell R Hartig T de Vries S Astell-Burt T Frumkin H. Green cities and health: a question of scale? J Epidemiol Community Health. (2012) 66:160–5. 10.1136/jech.2011.137240 22003083

[27] Pereira G Foster S Martin K Christian H Boruff BJ Knuiman M. The association between neighborhood greenness and cardiovascular disease: an observational study. BMC Public Health. (2012) 12:1. 10.1186/1471-2458-12-466 22720780 PMC3476430

[28] Roy S Byrne J Pickering C. A systematic quantitative review of urban tree benefits, costs, and assessment methods across cities in different climatic zones. Urban For Urban Green. (2012) 11:351–63. 10.1016/j.ufug.2012.06.006

[29] Seyler BC Gaoue OG Tang Y Duffy DC. Understanding knowledge threatened by declining wild orchid populations in an urbanizing China (Sichuan). Environ Conserv. (2019) 46:318–25. 10.1017/S0376892919000171

[30] Song W Pijanowski BC Tayyebi A. Urban expansion and its consumption of high-quality farmland in Beijing, China. Ecol Indic. (2015) 54:60–70. 10.1016/j.ecolind.2015.02.015

[31] Liu Y Fang F Li Y. Key issues of land use in China and implications for policy making. Land Use policy. (2014) 40:6–12. 10.1016/j.landusepol.2013.03.013

[32] Xu Z Zhang Z Li C. Exploring urban green spaces in China: spatial patterns, driving factors and policy implications. Land Use Policy. (2019) 89:104249. 10.1016/j.landusepol.2019.104249

[33] Peng J Zhao M Guo X Pan Y Liu Y. Spatial-temporal dynamics and associated driving forces of urban ecological land: a case study in Shenzhen City, China. Habitat Int. (2017) 60:81–90. 10.1016/j.habitatint.2016.12.005

[34] Chen B Nie Z Chen Z Xu B. Quantitative estimation of 21st-century urban greenspace changes in Chinese populous cities. Sci Total Environ. (2017) 609:956–65. 10.1016/j.scitotenv.2017.07.238 28783908

[35] Qian Y Zhou W Li W Han L. Understanding the dynamic of greenspace in the urbanized area of Beijing based on high resolution satellite images. Urban For Urban Green. (2015) 14:39–47. 10.1016/j.ufug.2014.11.006

[36] Song Y Huang B Cai J Chen B. Dynamic assessments of population exposure to urban greenspace using multi-source big data. Sci Total Environ. (2018) 634:1315–25. 10.1016/j.scitotenv.2018.04.061 29710631

[37] Song Y Chen B Kwan MP. How does urban expansion impact people's exposure to green environments? A comparative study of 290 Chinese cities. J Clean Prod. (2020) 246:119018. 10.1016/j.jclepro.2019.119018

[38] Jim CY Chen WY. Ecosystem services and valuation of urban forests in China. Cities. (2009) 26:187–94. 10.1016/j.cities.2009.03.003

[39] Chen X Pei T Zhou Z Teng M He L Luo M. Efficiency differences of roadside greenbelts with three configurations in removing coarse particles (PM < inf>10 < /inf>): a street scale investigation in Wuhan, China. Urban For Urban Green. (2015) 14:354–60. 10.1016/j.ufug.2015.02.013

[40] Ng E Chen L Wang Y Yuan C. A study on the cooling effects of greening in a high-density city: an experience from Hong Kong. Build Environ. (2012) 47:256–71. 10.1016/j.buildenv.2011.07.014

[41] Zhang B Xie G. di, Gao J xi, Yang Y. The cooling effect of urban green spaces as a contribution to energy-saving and emission-reduction: a case study in Beijing, China. Build Environ. (2014) 76:37–43. 10.1016/j.buildenv.2014.03.003

[42] Xing Y Brimblecombe P. Trees and parks as “the lungs of cities”. Urban Urban Green. (2020) 48:1–13. 10.1016/j.ufug.2019.126552

[43] Jin P Gao Y Liu L Peng Z Wu H. Maternal health and green spaces in China: a longitudinal analysis of MMR based on spatial panel model. Healthcare. (2019) 7:154. 10.3390/healthcare7040154 31810235 PMC6956252

[44] Liu L Zhong Y Ao S Wu H. Exploring the relevance of green space and epidemic diseases based on panel data in China from 2007 to 2016. Int J Environ Res Public Health. (2019) 16:2551. 10.3390/ijerph16142551 31319532 PMC6679052

[45] Zhao S Zhou D Zhu C Sun Y Wu W Liu S. Spatial and temporal dimensions of urban expansion in China. Environ Sci Technol. (2015) 49:9600–9. 10.1021/acs.est.5b00065 26212783

[46] Yue TX Wang YA Chen SP Liu JY Qiu DS Deng XZ. Numerical simulation of population distribution in China. Popul Environ. (2003) 25:141–63. 10.1023/B:POEN.0000015562.88915.01

[47] Seo A Yamamoto K Akai A Akagi D Takayama T Hoshina K. The relationship between medical expenses and the severity of peripheral arterial disease in Japan. Heart Vessels. (2018) 33:853–8. 10.1007/s00380-018-1127-3 29396768

[48] Akhavan Hejazi SM Mazlan M Abdullah SJF Engkasan JP. Cost of post-stroke outpatient care in Malaysia. Singapore Med J. (2015) 56:116–9. 10.11622/smedj.2015025 25715857 PMC4350462

[49] Jiang C. 温江区苗木经纪的调查报告 Wēnjiāng qu miáomù jingjì de diàochá bàogào. Chengdu, China: Southwestern University of Finance and Economics (2011).

[50] Du K. 基于产业集群视角下的温江花木产业研究 Jiyú chǎnyè jíqún shìjiǎo xià de wēnjiāng huāmù chǎnyè yánjiu. Ya'an, China: Sichuan Agricultural University (2012).

[51] Statistical Bureau of Sichuan. Sichuan Statistical Yearbook. (2017). Available online at: http://web.sctjj.cn/tjcbw/tjnj/2017/zk/indexeh.htm (accessed February 14, 2020).

[52] NASA. MOD13Q1—MODIS/Terra Vegetation Indices 16-Day L3 Global 250m SIN Grid. LAADS DAAC. (2016). Available online at: https://ladsweb.modaps.eosdis.nasa.gov/missions-and-measurements/products/MOD13Q1/ (accessed February 14, 2020).

[53] R Core Team,. R: A Language Environment for Statistical Computing. (2019). Available online at: https://www.r-project.org/ (accessed June 26, 2020).

[54] NCHS. ICD-10-CM Official Guidelines for Coding and Reporting FY 2020. National Center for Health Statistics. (2020). p. 1–121.

[55] Voeks RA Leony A. Forgetting the forest: assessing medicinal plant erosion in Eastern Brazil. Econ Bot. (2004) 58:94–106. 10.1663/0013-0001(2004)58[S294:FTFAMP]2.0.CO;2

[56] Laffitte B Seyler BC Tang Y. Environmental education to engage urban youth: anecdotes from Southwest China Southwest China. J Biol Educ. (2022) 1–13. 10.1080/00219266.2022.2118812

[57] Hazer M Formica MK Dieterlen S Morley CP. The relationship between self-reported exposure to greenspace and human stress in Baltimore, MD. Landsc Urban Plan. (2018) 169:47–56. 10.1016/j.landurbplan.2017.08.006

[58] Peters K Elands B Buijs A. Social interactions in urban parks: Stimulating social cohesion? Urban For Urban Green. (2010) 9:93–100. 10.1016/j.ufug.2009.11.003

[59] Rasidi MH Jamirsah N Said I. Urban green space design affects urban residents' social interaction. Proc Soc Behav Sci. (2012) 68:464–80. 10.1016/j.sbspro.2012.12.242

[60] de Vries S van Dillen SME Groenewegen PP Spreeuwenberg P. Streetscape greenery and health: Stress, social cohesion and physical activity as mediators. Soc Sci Med. (2013) 94:26–33. 10.1016/j.socscimed.2013.06.030 23931942

[61] Dadvand P Tischer C Estarlich M Llop S Dalmau-Bueno A López-Vicente M. Lifelong residential exposure to green space and attention: a population-based prospective study. Environ Health Perspect. (2017) 125:1–8. 10.1289/EHP694 28934095 PMC5915181

[62] Bratman GN Daily GC Levy BJ Gross JJ. The benefits of nature experience: Improved affect and cognition. Landsc Urban Plan. (2015) 138:41–50. 10.1016/j.landurbplan.2015.02.005

[63] Cavanagh JAE Zawar-Reza P Wilson JG. Spatial attenuation of ambient particulate matter air pollution within an urbanised native forest patch. Urban For Urban Green. (2009) 8:21–30. 10.1016/j.ufug.2008.10.002

[64] Gidlöf-Gunnarsson A Öhrström E. Noise and well-being in urban residential environments: The potential role of perceived availability to nearby green areas. Landsc Urban Plan. (2007) 83:115–26. 10.1016/j.landurbplan.2007.03.003

[65] Berry R Livesley SJ Aye L. Tree canopy shade impacts on solar irradiance received by building walls and their surface temperature. Build Environ. (2013) 69:91–100. 10.1016/j.buildenv.2013.07.009

[66] Wang X Yang H Duan Z Pan J. Spatial accessibility of primary health care in China: a case study in Sichuan Province. Soc Sci Med. (2018) 209:14–24. 10.1016/j.socscimed.2018.05.023 29778934

[67] Wang X Seyler BC Han W Pan J. An integrated analysis of spatial access to the three-tier healthcare delivery system in China: a case study of Hainan Island. Int J Equity Health. (2021) 20:1–15. 10.1186/s12939-021-01401-w 33579289 PMC7881625

[68] Hartig T Mitchell R De Vries S Frumkin H. Nature and health. Annu Rev Public Health. (2014) 35:207–28. 10.1146/annurev-publhealth-032013-182443 24387090

[69] Fan P Xu L Yue W Chen J. Accessibility of public urban green space in an urban periphery: the case of Shanghai. Landsc Urban Plan. (2017) 165:177–92. 10.1016/j.landurbplan.2016.11.007

[70] Zhong J Li Z Sun Z Tian Y Yang F. The spatial equilibrium analysis of urban green space and human activity in Chengdu, China. J Clean Prod. (2020) 259:120754. 10.1016/j.jclepro.2020.120754

[71] Cao Q Liang Y Niu X. China's air quality and respiratory disease mortality based on the spatial panel model. Int J Environ Res Public Health. (2017) 14:1081. 10.3390/ijerph14091081 28927016 PMC5615618

[72] Zeng Y Cao Y Qiao X Seyler BC Tang Y. Air pollution reduction in China: recent success but great challenge for the future. Sci Total Environ. (2019) 663:329–37. 10.1016/j.scitotenv.2019.01.262 30711599

[73] Ren Y Qu Z Du Y Xu R Ma D Yang G. Air quality and health effects of biogenic volatile organic compounds emissions from urban green spaces and the mitigation strategies. Environ Pollut. (2017) 230:849–61. 10.1016/j.envpol.2017.06.049 28734266

[74] Liu L Seyler BC Liu H Zhou L Chen D Liu S. Biogenic volatile organic compound emission patterns and secondary pollutant formation potentials of dominant greening trees in Chengdu, southwest China. J Environ Sci. (2022) 114:179–93. 10.1016/j.jes.2021.08.033 35459483

